# Assessment of Arterial Stiffness by Cardio-Ankle Vascular Index for Prediction of Five-Year Cardiovascular Events After Coronary Artery Bypass Surgery

**DOI:** 10.5334/gh.1053

**Published:** 2021-12-27

**Authors:** Alexei N. Sumin, Anna V. Shcheglova, Irina I. ZHidkova, Sergey V. Ivanov, Olga L. Barbarash

**Affiliations:** 1 Laboratory of Comorbidity in Cardiovascular Diseases, Research Institute for Complex Issues of Cardiovascular Diseases, Kemerovo, Russian Federation, RU; 2 Circulatory Pathology Laboratory, Research Institute for Complex Issues of Cardiovascular Diseases, Kemerovo, Russian Federation, RU; 3 Laboratory of Endovascular and Reconstructive Surgery of the Heart and Vessels, Research Institute for Complex Issues of Cardiovascular Diseases, Kemerovo, Russian Federation, RU; 4 Research Institute for Complex Issues of Cardiovascular Diseases, Kemerovo, Russian Federation, RU

**Keywords:** arterial stiffness, cardio-ankle vascular index, coronary artery bypass surgery, five-year follow-up

## Abstract

The study aim was to investigate the possibility of cardiovascular complications development predicting during a five-year follow-up of patients after coronary artery bypass grafting (CABG) using the cardio-ankle vascular index (CAVI) assessment.

**Methods:**

Three hundred and fifty–six patients after elective CABG were enrolled in the study. Prior to surgery, arterial stiffness was assessed in all patients using CAVI. The follow-up was performed five years after the surgery, information was obtained on 238 patients, who were divided into two groups: patients with pathological (≥9.0, n = 88), and normal (<9.0, n = 150) CAVI.

**Results:**

Pathological CAVI (≥9.0) was detected in 33% patients before CABG, in stepwise analyses only age and left atrium dimensions statistically significantly predicted CAVI. In patients with pathological CAVI the combined endpoint (major adverse cardiovascular events and hospitalization) and cardiovascular death developed more often in a five-year follow-up after CABG compared with normal CAVI (48.86% versus 34.9%, p = 0.034 and 4.55% versus 0.67%, p = 0.049, respectively). Pathological CAVI (p = 0.021) and the number of coronary bypass grafts (p = 0.023) were independent factors associated with the combined endpoint.

**Conclusions:**

Patients with pathological CAVI before CABG surgery are more likely to develop cardiovascular complications and cardiovascular death within a subsequent five-year follow-up. Evaluation of CAVI after CABG in dynamics deserves further study, it is important for monitoring the effects of secondary prevention and the possibility of influencing the prognosis.

## 1. Introduction

Arterial stiffness is an integral indicator reflecting the risk factors influence on the vascular wall state. This parameter is used in a clinical setting, as well as for additional risk assessment in population samples [1]. Nevertheless, the traditional indicator of arterial stiffness (carotid-femoral pulse wave velocity – cfPWV) has a number of disadvantages that complicate its practical use – dependence on blood pressure level, lack of measurement standardization, inconvenience for the patient and staff [1]. To overcome these limitations, a new indicator has been proposed and has been used for more than 10 years – the cardio-ankle vascular index (CAVI), which is based on the stiffness parameter β assessment [2]. This measure reflects the arterial stiffness of the local arterial segment and is not influenced by blood pressure during measurement. Accordingly, CAVI is less dependent on the blood pressure level, is more suitable for assessment in dynamics, and is more convenient for determination in a clinical setting. Since CAVI evaluates other segments of the vascular bed (in contrast to cfPWV) – from the ascending aorta to the tibial artery, it reflects the state of not only elastic, but also muscular arteries, which can potentially affect its diagnostic capabilities. Therefore, studies are continuing to investigation the CAVI clinical and prognostic value.

In a recent review by Saiki A. et al. [3], showed that higher CAVI values were found in atherosclerosis of various localization, in patients with coronary artery disease, in stroke, in chronic kidney disease, in the presence of the intima-media complex thickening in the carotid arteries. In addition, CAVI was high in patients with risk factors for cardiovascular diseases: arterial hypertension, diabetes mellitus, dyslipidemia, hyperuricemia, obstructive apnea syndrome, and smoking. As well, metabolic syndrome, sarcopenia, cognitive impairment in old age, and a high level of daily stress are associated with an increase in CAVI [3].

In prospective studies among individuals at high risk of cardiovascular disease (presence of hypertension, diabetes, obesity, chronic kidney disease), baseline CAVI was a predictor of future cardiovascular events, the meta-analyses showed a moderate association between CAVI and episodes of cardiovascular disease [4]. In patients with coronary artery disease, the prognostic value of CAVI is still poorly understood. Thus, in patients with acute coronary syndrome (ACS), an increase in CAVI was an independent predictor of cardiovascular events and death from cardiovascular diseases [5,6]. Similar results were obtained in patients with stable coronary artery disease [7], when observing patients for a year after coronary artery bypass grafting [8]. Nevertheless, the assessment of CAVI has not yet found its place in patients with coronary artery disease; apparently, further research is required on its clinical and prognostic significance, including in diverse clinical situations and different geographic regions.

This served as the basis for the present study, the purpose of which was to investigate the possibility of cardiovascular complications development predicting during a five-year follow-up of patients after coronary artery bypass grafting (CABG) using the CAVI assessment.

## 2. Subjects, Materials and Methods

### Study population

This single-center, observational study was conducted at the Federal State Budgetary Institution ‘Research Institute for Complex Issues of Cardiovascular Diseases.’ All patients who underwent CABG in the cardiovascular surgery department of the clinic from March 2012 to March 2013 were included. Patients with recent acute coronary syndrome, emergency coronary bypass grafting, valvular disease, and left ventricular ejection fraction ≤30% were not included. In addition, patients with ankle-brachial index value ≤0.9, presence of atrial fibrillation, atrial flutter at the time of the study, installed pacemaker, and high amputation of the lower extremities were excluded. The study protocol was approved by the Local Ethics Committee of the Federal State Budgetary Institution ‘Research Institute for Complex Issues of Cardiovascular Diseases.’ Patients were included in the study after they provided written informed consent.

### Data collection

Demographic and perioperative patient data were obtained from the electronic database of the institute’s CABG registry (certificate No. 2012020868 on registration of CABG database). For each patient who met the inclusion criteria, the following data were collected: age, sex, body mass index, present or absent history of myocardial infarction, hypertension, stroke, diabetes mellitus, heart failure, PCI, CABG, peripheral artery stenosis, dyslipidemia, smoking, creatinine and glucose levels. In addition, intraoperative parameters were recorded, including use of cardiopulmonary bypass; duration of surgery; aortic clamping time; number of grafts; and combined surgeries (ventriculoplasty, thrombectomy, radiofrequency ablation, carotid endarterectomy).

### Echocardiography

It was performed on an expert class apparatus ‘Vivid-7 Dimension’ (General Electric, USA) by two-dimensional echocardiography, Doppler echocardiography in pulsed and continuous wave mode, color Doppler scanning (CDS) in accordance with the current [9]. Diameter and thickness of the LV walls were measured in the two-dimensional M-mode. Left heart analysis included assessment of the size and volumetric parameters of the left ventricle, left ventricular mass, the maximum transverse diameter of the left atrium in the diastole (LA). Ejection fraction of the left ventricle (LVEF) was calculated according to the Simpson method.

Left ventricular diastolic parameters were studied using pulsed-wave Doppler imaging, including peak velocity of early (E) and late (A) transmitral filling of the left ventricle and their ratio (E/A) and the isovolumetric relaxation time of the left ventricle. The study was carried out within the first two days from the moment the patient was admitted to the hospital.

### Duplex ultrasonography assessment

The study was carried out on an expert class ultrasound device ‘Vivid 7 Dimension’ (General Electric, USA). Measurement of the thickness of the intima-media complex was performed in the common carotid artery. The patency of the vessel, the state of the vessel lumen, and the structure of the plaque were assessed. Doppler and B-mode according to the degree of stenosis measured the assessment of the narrowing of the arteries. The criteria for categorizing carotid artery stenosis were defined as: mild stenosis (30–49%); moderate and severe stenosis (50% and more).

Duplex ultrasonography of the lower extremity arteries, from common femoral to pedal arteries was performed for each patient using an expert class ultrasound device ‘Vivid 7 Dimension’ (General Electric, USA) and a 7.5 MHz linear-array transducer. The test was conducted by an experienced echocardiographer and followed standard imaging protocols. Examination was done with the patient lying supine after resting for at least 15 min. The common femoral and anterior tibial arteries were imaged with the patient in the supine position while those of the popliteal, peroneal and posterior tibial arteries were done in lateral position. The severity of stenosis was graded as follows: 30–49% – mild stenosis; 50% and more – moderate stenosis.

Multifocal atherosclerosis was established in the presence of subclinical lesions of peripheral arteries (stenosis 30% or more, or 50% or more).

### Measurement of CAVI

All patients additionally assessed the stiffness of the peripheral arteries – cardio-ankle vascular index (CAVI) using a VaSera VS-1000 device (Fukuda Denshi, Japan) according to the previously described method [10]. This device is a portable sphygmomanometer, four blood pressure cuffs were placed on the limbs; ECG electrodes were attached to the wrists. A phonocardiographic microphone was installed in the second intercostal space on the left. The CAVI index reflects the rigidity of the entire arterial segment. This index is calculated by the device automatically on the right and left (R-CAVI and L-CAVI) and originates from the so-called stiffness parameter ß in combination with a modified the Bramwell-Hill equation CAVI = a {(2p/ΔP) · ln (Ps/Pd) PWV2} + ß, which estimates the relationship between the speed of propagation of the pulse wave and the elasticity of the vascular wall. Considering CAVI, the measurement uses the mean BP of the brachial artery. In addition, when assessing CAVI, the stiffness parameter ß is taken into account, which is defined as the ratio of the natural logarithm of pressure (In [Ps/Pd]) to the degree of change in the inner diameter (D/ΔD) of the vessel. It is known that this parameter does not depend on internal pressure, and the higher ß, the lower the extensibility, and the greater the vascular stiffness.

At the same time, the ankle-brachial index was assessed. With ABI values less than 0.9, the patient was excluded from the study. With a CAVI value ≥9.0 on at least one side, the index was considered pathological.

### Follow-up

Patients included in the study for five years were observed by a cardiologist in their residence place. The follow-up was performed five years after the surgery by telephone surveys. We managed to find out information about the health status of 238 (65.2%) patients. To assess the prognosis, we analyzed coronary and non-coronary death, myocardial infarction, acute cerebrovascular accident/transient ischemic attack, percutaneous coronary intervention, carotid endarterectomy, pulmonary embolism, hospitalizations for cardiovascular diseases. The primary endpoint included all of these outcomes (combined endpoint). Secondary endpoints included cardiovascular and total mortality, nonfatal myocardial infarction, and nonfatal stroke.

### Statistical analyses

Standard Statistica 8.0 (Tulsa, OK) and SPSS, Version 17.0 (SPSS Inc, Chicago, IL) software were used for statistical analyses. Baseline data and follow-up CABG outcomes were assessed in patients with normal and pathological CAVI values. Qualitative values were presented in absolute numbers (n) and percentage (%), and comparisons between the groups were performed using chi-square tests. The normality of the distribution was verified using the Kolmogorov-Smirnov test. For a distribution other than normal, all quantitative variables were presented as the median and quartiles (lower quartile, upper quartile). When comparing the quantitative data between the two groups, the Mann-Whitney test was used.

Multiple linear regression analysis (enter method and stepwise selection) was applied to evaluate significance correlation of CAVI with preoperative parameters in patients. Performance of preoperative indices for diagnosing the pathological CAVI presence was evaluated through receiver operating characteristic curve analysis.

Logistic regression analysis was performed to estimate the odds ratio (OR) and 95% confidence interval (CI) for the effect of possible factors on the primary outcome (the combined endpoint development – total mortality, nonfatal MI, and nonfatal stroke, re-revascularization and hospitalization due to relapse or progression of angina pectoris). Binary logistic regression analysis (Forward LR method) was performed to estimate the effect of independent factors on the primary outcome. P < 0.05 was considered statistically significant.

## 3. Results

### Patient baseline characteristics

**Figure 1:**
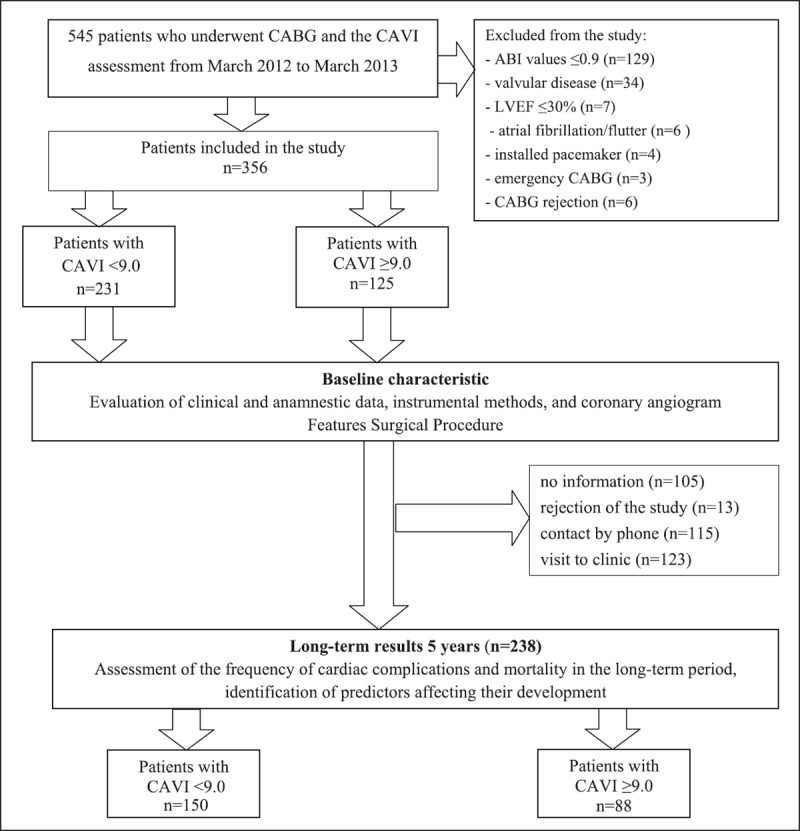
Flowchart of persons selection. CAVI: cardio-ankle vascular index. ABI – ankle-brachial index; LVEF – left ventricular ejection fraction; CABG – coronary artery bypass graft; CAVI – cardio-ankle vascular index.

A flow diagram of study participants is presented in Figure 1. During the period from March 2012 to March 2013, 545 patients were admitted to the research institute’s clinic for elective CABG. Exclusion criteria included recent acute coronary syndrome (n = 3); installed pacemaker (n = 4), valvular disease (n = 34), LVEF <30% (n = 7); ABI < 0.9 (n = 129), persistent atrial fibrillation/flutter (n = 6); and CABG rejection (n = 6). A total of 356 patients were included in the study. When evaluating the five-year results, it was not possible to obtain information on 105 patients, 13 patients refused to participate in the study, information was obtained on 238 patients (115 patients – contact by phone, 123 – visit to the clinic). Further analysis was carried out in 238 patients, divided into groups with normal (n = 150) and pathological (n = 88) CAVI. Among patients without information about the five-year follow-up stage, the number of patients with pathological CAVI was comparable to the analyzed group (33% and 37%). When comparing the initial data among those who dropped out at the five-year stage in the groups with normal and pathological CAVI (Supplement Table 1), they were similar among those observed in the analyzed group (Table 1).

**Table 1:** Baseline characteristics of the study groups (n = 238).

Variables	Group 1 CAVI < 9.0 n = 150	Group 2 CAVI ≥ 9.0 n = 88	p

Age. years	56.5 (51.0; 62.0)	62.0 (55.5; 68.5)	<0.001
Male sex (n. %)	119 (79.33)	64 (72.73)	0.243
Height (cm)	170 (164.0; 176.0)	170.0 (164.0; 175.5)	0.834
Weight (kg)	80.0 (71.0; 89.0)	80.0 (73.5; 86.5)	0.841
BMI (kg/m2)	28.39 (25.2; 31.83)	28.36 (26.63; 30.48)	0.752
Smoking (n. %)	45 (30.0)	18 (20.45)	0.011
Myocardial infarction history (n. %)	100 (66.67)	52 (59.09)	0.240
Hypertension (n. %)	122 (81.33)	82 (93.18)	0.011
Stroke history (n. %)	10 (6.8)	6 (6.82)	0.964
Transischemic attack (n. %)	1 (0.67)	1 (1.14)	0.701
Diabetes mellitus (n. %)	14 (9.33)	20 (22.73)	0.0043
PCI history (n. %)	14 (9.33)	7 (7.95)	0.717
CABG history (n. %)	1 (0.67)	1 (1.14)	0.701
Carotid endarterectomy (n. %)	3 (2.0)	2 (2.27)	0.887
No angina (n. %)	24 (16.0)	24 (27.3)	0.038
Angina I functional class (n. %)	6 (4.0)	2 (2.27)	0.47
Angina II functional class (n. %)	54 (36.0)	25 (28.41)	0.216
Angina III functional class (n. %)	63 (42.0)	33 (37.5)	0.468
Angina IV functional class (n. %)	3 (2.0)	4 (4.55)	0.129
HF NYHA I (n. %)	91 (60.6)	50 (56.8)	0.559
HF NYHA II (n. %)	45 (30.0)	33 (37.5)	0.234
HF NYHA III (n. %)	5 (3.33)	3 (3.41)	0.975
Surgical procedure			
Cardiopulmonary bypass (n. %)	124 (82.67)	77 (87.5)	0.321
Bypass graft number	2.5 (2.0; 3.0)	3.0 (2.0; 3.0)	0.257
Cardiopulmonary bypass duration (min)	98.0 (77.0; 112.0)	94.0 (79.0; 107.0)	0.645
Оperation duration (min)	243.0 (198.0; 300.0)	240.0 (79.0; 107.0)	0.691
Ventriculoplasty. n (%)	8 (5.33)	3 (3.41)	0.494
Thrombectomy. n (%)	5 (3.33)	0	0.083
Radiofrequency ablation. n (%)	2 (1.33)	2 (2.27)	0.586
Carotid endarterectomy. n (%)	3 (2.0)	2 (2.27)	0.887
Preoperative echocardiogram			
LV EDD (cm)	109.0 (103.0; 118.0)	109.0 (103.0; 116.0)	0.871
LV ESD (cm)	116.0 (111.0; 123.0)	114.0 (109.0; 124.0)	0.442
LV EDV (ml)	136.0 (123.5; 167.0)	131.0 (117.0; 157.0)	0.161
LV ESV (ml)	141.0 (127.0; 156.0)	142.0 (122.0; 153.0)	0.779
LA (mm)	108.0 (102.0; 113.0)	109 (103.0; 116.0)	0.117
LV EF (%)	55.0 (48.0; 59.0)	53.0 (48.0; 59.0)	0.822
LVMMI (g/m^2^)	151.25 (124.8; 177.7)	157.4 (138.9; 194.15)	0.068
E (cm/s)	52.0 (47.0; 66.0)	49.0 (40.0; 71.0)	0.670
А (cm/s)	56.0 (0.88; 73.0)	58 (46.0; 70.0)	0.835
E/A ratio	0.87 (0.7; 1.2)	0.775 (0.67; 1.2)	0.195
DT (ms)	201.0 (197.0; 243.0)	209.0 (198.0; 230.0)	0.834
IVRT (ms)	102.0 (91.0; 113.0)	104.0 (90.0; 116.0)	0.784
Laboratory data			
Total cholesterol (mmol/L)	4.8 (3.8; 5.8)	4.25 (3.75; 5.25)	0.189
HDL cholesterol (mmol/L)	1.22 (0.94; 1.49)	1.24 (1.05; 1.58)	0.231
LDL cholesterol (mmol/L)	2.84 (1.97; 3.79)	2.5 (1.88; 3.7)	0.175
Triglycerides (mmol/L)	1.55 (1.1; 2.1)	1.46 (0.96; 1.7)	0.140
Atherogenicity index	2.43 (1.9; 3.3)	2.9 (2.1; 4.5)	0.048
Creatinine (µmol/L)	77.0 (64.0; 95.0)	75.0 (65.0; 95.0)	0.948
Glucose (mmol/L)	5.8 (5.26; 6.6)	5.6 (4.9; 6.5)	0.605
Сoronary angiography			
1-coronary artery disease (n. %)	37 (24.66)	20 (22.73)	0.606
2-coronary artery disease (n. %)	51 (34.0)	25 (28.41)	0.329
3-coronary artery disease (n. %)	62 (41.33)	43 (48.86)	0.288
LMCA ≥ 50% (n. %)	37 (24.67)	18 (20.45)	0.456
Lesion of non-coronary arteries			
Сarotid artery stenoses ≥30% (n. %)	25 (16.67)	18 (20.45)	0.463
Сarotid artery stenoses ≥50% (n. %)	16 (10.67)	14 (15.91)	0.239
Сarotid artery stenoses on both sides ≥30% (n. %)	14 (9.33)	14 (15.91)	0.128
Lower extremities arteries stenoses ≥30% (n. %)	17 (11.33)	9 (10.23)	0.791
Lower extremities arteries stenoses ≥50% (n. %)	5 (3.33)	5 (5.68)	0.383
Lower extremities arteries stenoses on both sides ≥30% (n. %)	15 (10.0)	8 (9.09)	0.818
CIMT (mm)	1.1 (1.0; 1.2)	1.2 (1.0; 1.2)	0.278

*Note*: Continuous data are presented as median (lower quartile. upper quartile). Abbreviations: EDD – end-diastolic dimension; ESD end-systolic dimension; DT – deceleration time; IVRT – isovolumic relaxation time; LVEDV left ventricular end-diastolic volume; LV ESV – left ventricular end-systolic volume; LA – left atrium; CABG – coronary artery bypass graft; LV EF – left ventricular ejection fraction; NYHA – New York Heart Association; PCI – percutaneous coronary intervention; BMI – body mass index; LDL – low-density lipoproteins; CIMT – carotid intima-media thickness; HDL – high-density lipoproteins; LVMI – left ventricular mass index; LMCA – left main coronary artery.

**Figure 2:**
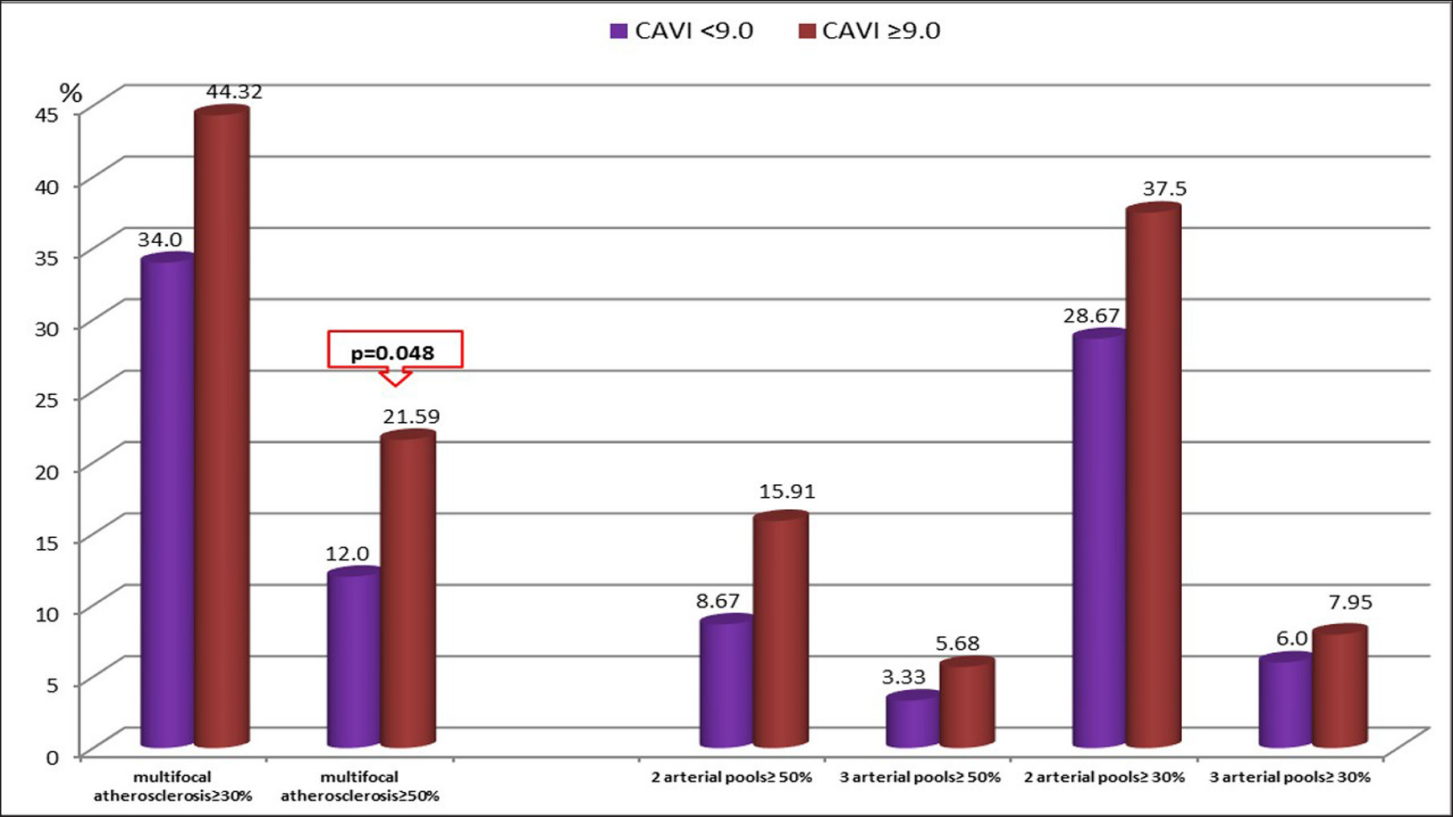
Prevalence of multifocal atherosclerosis in groups with abnormal and normal CAVI. CAVI – cardio-ankle vascular index.

Comparison of demographic data in patients with pathological and normal CAVI showed no gender differences between the groups (p = 0.243); men predominated in both groups. Patients in the group with pathological CAVI were older (p < 0.001), they more often had arterial hypertension (p = 0.011) and diabetes mellitus (p = 0.0043), but less often the prevalence of smoking (p = 0.0107) compared to the group with normal CAVI. Also, in this group, patients without angina pectoris (p = 0.038) and with the presence of multifocal atherosclerosis (p = 0.048) were more often detected (Table 1, Figure 2). For the remaining preoperative parameters (blood biochemical parameters, data on systolic and diastolic left ventricular function, the number of coronary arteries lesions, the severe coronary and heart failure presence) no significant differences were found between groups. We also did not reveal any intergroup differences according to the perioperative data (duration of operations, number of shunts, and frequency of operations with cardiopulmonary bypass). In the group with pathological CAVI, the atherogenic index was higher (p = 0.048).

### Relationship between CAVI and preoperative parameters

Multiple regression was run to predict CAVI based on preoperative parameters: age, echocardiographic parameters, the presence of comorbidities, subclinical peripheral arterial lesions, BMI, blood biochemical parameters, the severity of coronary and heart failure, and the number of coronary arteries affected. In the model with the inclusion of all variables (Supplement Table 2) age (B = 0.070, p < 0.001), the left atrium dimensions (B = 0.647, p < 0.001), two-vessel CAD (B = 0.442, p = 0.027), and three-vessel coronary artery disease (B = 0.276, p = 0.032) were associated with CAVI. In stepwise analyses only age and left atrium dimensions statistically significantly predicted CAVI, F (5, 1162) = 82.622, p < 0.0001, R2 = 0.263 (Table 2).

**Table 2:** Linear regression analysis (stepwise method) for the relationship of CAVI with other variables (Coefficients)^a^.

Model		Unstandardized Coefficients		Standardized Coefficients	t	Sig.

		B	Std. Error	Beta		

1	(Constant)	3.816	0.634		6.015	0.000
	Age	0.081	0.011	0.421	7.633	0.000
2	(Constant)	1.744	0.788		2.213	0.028
	Age	0.073	0.011	0.378	6.939	0.000
	Left atrium	0.602	0.143	0.230	4.212	0.000

^a^ Dependent Variable: CAVI.

**Figure 3:**
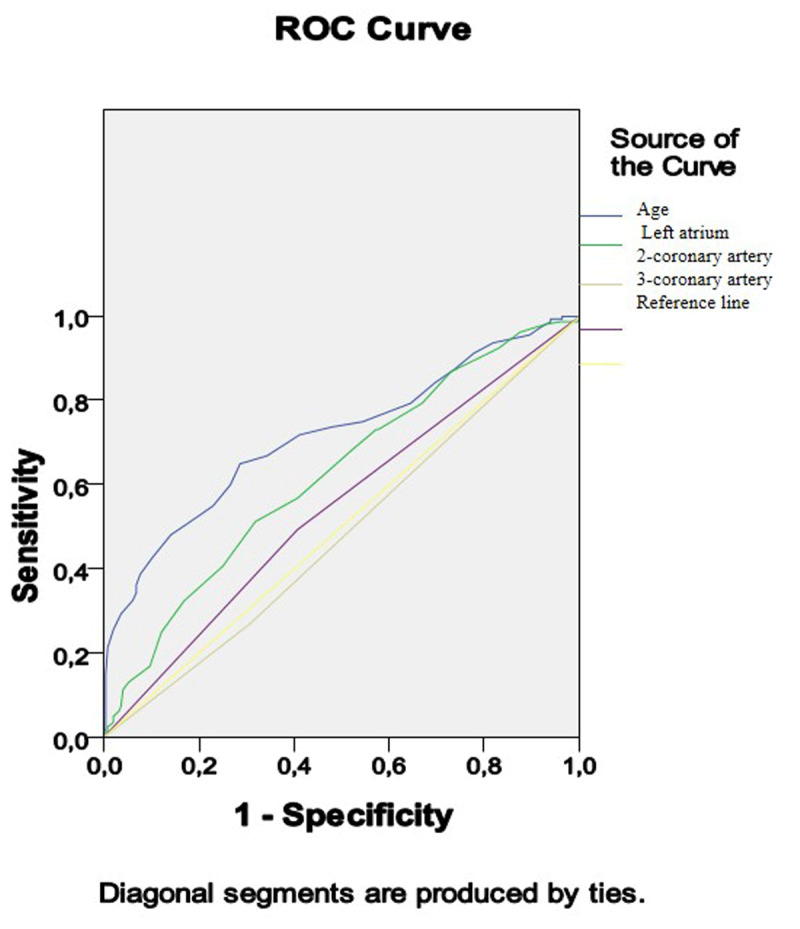
Receiver operating characteristic curve analysis. Performance of baseline parameters in discriminating of the pathological CAVI. CAVI – cardio-ankle vascular index.

The association of preoperative parameters (age, LA dimensions, two- and three-vessel CAD) with the pathological CAVI presence is presented in Figure 3. As shown in Table 3, the areas under the curves were only > 0.7 for age, indicating acceptable discrimination ability. The areas under the curves for the remaining indicators were <0.7, which indicated an unacceptable ability to distinguish. Three-vessel CAD was associated with pathological CAVI, and two-vessel disease – with normal CAVI.

**Table 3:** Receiver operating characteristic curve analysis. Performance of baseline parameters in discriminating of the pathological CAVI. Area under the curve.

Test Result Variable(s)	Area	Std. Error^a^	Asymptotic Sig.	Asymptotic 95% Confidence Interval	

				Lower Bound	Upper Bound

Age	0.716	0.027	0.000	0.663	0.769
Left atrium dimention	0.624	0.028	0.000	0.569	0.679
2-coronary artery disease	0.481	0.029	0.520	0.424	0.538
3-coronary artery disease	0.543	0.029	0.140	0.486	0.601

The test result variable(s): Age. Left atrium dimention. two-coronary artery disease. three-coronary artery disease has at least one tie between the positive actual state group and the negative actual state group. ^a^ Under the nonparametric assumption. ^b^ Null hypothesis: true area = 0.5.

### Five-year follow-up CABG adverse events in patients with pathological and normal CAVI

**Figure 4:**
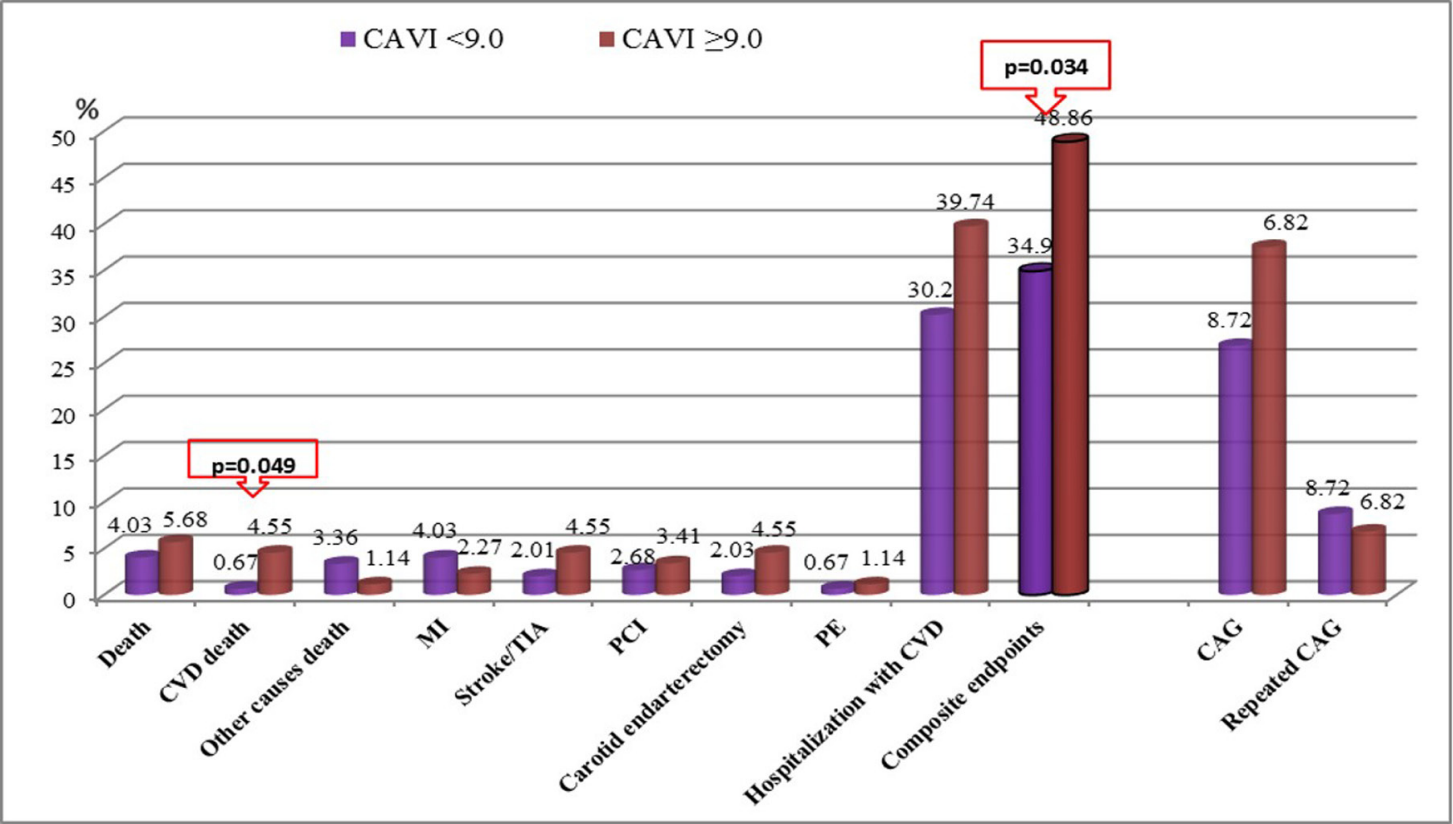
Structure of complications in the long-term period after CABG. CVD – cardiovascular disease; MI – myocardial infarction; TIA – transient ischemic attack; PCI – percutaneous coronary intervention; PE – pulmonary embolism; CAG – coronary angiography; CAVI – cardio-ankle vascular index.

The development of the primary endpoint (combined endpoint – death, stroke/TIA, PCI, CEE, PE, hospitalization for cardiovascular diseases) was more often observed in patients with pathological CAVI compared with patients with normal CAVI (48.86% and 34.9%, respectively, p = 0.034) (Figure 4). Among the secondary endpoints, death from all causes was equally common in both groups (p = 0.389), death from cardiac causes was more common in the group with pathological CAVI (4.55%) than in the group with normal CAVI (0.67%, p = 0.049). There were no statistically significant differences for other adverse cardiovascular events.

### Logistic regression analyses

In the univariate analyses (Table 4), the OR for the development of combined endpoint in patients with pathological CAVI compared with patients with normal CAVI was 1.78 (95% CI 1.04–3.06; p = 0.031). The using three bypass grafts as a single predictor predicted a lower risk of CEP, with an OR of 0.8 (95% CI 0.67–0.96; p = 0.015). According to the results of multivariate analysis, the same factors remained independent predictors of the development of CEP: the pathological CAVI presence worsened the prognosis (OR 1.88; 95% CI 1.04–3.4, p = 0.033), and the using three bypass grafts improved it (OR 0.79; 95% CI 0.65–0.95, p = 0.013).

**Table 4:** Factors associated with the combined end point development within five years after CABG according to logistic regression analysis.

Variables	OR (95% CI)	p Value

Univariate logistic regression analysis		

CAVI ≥9.0	1.78 (1.04–3.06)	0.031
Male sex	0.58 (0.32–1.07)	0.08
Age	0.97 (1.04–0.97)	0.670
Myocardial infarction history	0.89 (0.34–2.34)	0.824
Stroke history	1.81 (0.67–4.91)	0.236
Hypertension	1.49 (0.69–3.21)	0.307
Diabetes mellitus	1.66 (0.8–3.4)	0.169
Atrial fibrillation	0.98 (0.97–1.0)	0.058
Smoking	0.87 (0.48–1.57)	0.652
Left main coronary artery ≥50%	0.67 (0.35–1.2)	0.215
3-coronary artery disease	1.0 (0.83–1.2)	0.96
1 – bypass graft	1.54 (0.8–2.9)	0.187
2 – bypass graft	1.28 (0.96–1.7)	0.085
3 – bypass graft	0.8 (0.67–0.96)	0.015
Multifocal atherosclerosis ≥30%	1.53 (0.9–2.6)	0.111
Multifocal atherosclerosis ≥50%	1.58 (0.79–3.17)	0.188
Сarotid artery stenosis ≥30% ≥30%	1.28 (0.65–2.5)	0.466
Сarotid artery stenosis ≥50%	1.78 (0.83–3.81)	0.134
Stenosis of the arteries lower extremities on both sides ≥30%	2.08 (0.95–4.59)	0.065
Multivariable logistic models Adjusted for age and sex. p < 0.001		

3 – bypass graft	0.79 (0.65–0.95)	0.013
CAVI ≥9.0	1.88 (1.04–3.4)	0.033

In multiple binary logistic regression model (forward LR method) the following factors had a significant association (χ2(2) = 9.94, p = 0.007) with the combined endpoint – pathological CAVI (B = 0.666, p = 0.021) and bypass grafts number (B = –0.364, p = 0.023), and this model explained only 20.0% (Nagelkerke R^2^) of the variance in CEP and correctly classified 62.6% of cases (Table 5).

**Table 5:** Results of binary logistic regression (forward LR method): association of factors with the risk of combined end point development.

Variables in the Equation							

		B	S.E.	Wald	df	Sig.	Exp(B)

Step 1^a^	CAVI ≥9.0	0.615	0.285	4.667	1	0.031	1.850
	Constant	–0.590	0.175	11.420	1	0.001	0.554
Step 2^b^	Graft number	–0.364	0.161	5.137	1	0.023	0.695
	CAVI ≥9.0	0.666	0.290	5.287	1	0.021	1.946
	Constant	0.249	0.404	0.380	1	0.538	1.283

^a^ Variable(s) entered on step 1: CAVI ≥9.0. ^b^ Variable(s) entered on step 2: Graft number.

## 4. Discussion

In the present study, for the first time it was shown that in patients with pathological CAVI the combined endpoint and cardiovascular death developed more often in a five-year follow-up after CABG. Pathological CAVI and the number of coronary bypass grafts were independent factors associated with the combined endpoint.

In our previous work, the effect of CAVI on the annual prognosis after CABG was assessed. The presence of pathological CAVI was associated with a higher incidence of the combined endpoint (death, myocardial infarction, stroke, hospitalization, angina pectoris), which persisted in multivariate analysis (OR 2.5; 95% CI 1.26–5.08, p = 0.008) [8]. In other studies, the predictive value of pathological CAVI was studied in CAD patients without surgical treatment [5,6,7]. In patients with stable CAD, the incidence of cardiovascular events after 2.9 years in patients with initially elevated CAVI was significantly higher in the group without improvement in CAVI than in the group with its improvement [7]. When observing patients with ACS for 1.25 years, the presence of CAVI > 8.325 in patients was an independent predictor of cardiovascular events and nonfatal ischemic strokes [5]. Kirigaya J et al. [6], when observing patients with acute coronary syndrome for five years, a significantly higher probability of MACE (cardiovascular death, recurrence of ACS, heart failure requiring hospitalization, or stroke) was demonstrated in the group with high CAVI than in the group with low CAVI (p < 0.001). In multivariate analysis, CAVI was an independent predictor of MACE and death from cardiovascular disease [6]. These results are similar to our study in terms of duration of observation and significant endpoints.

A recent study showed that CAVI is one of the independent determinants of the hyperemic microvascular resistance index in patients with non-obstructive coronary artery disease, that is, it is associated with coronary microvascular dysfunction [11]. It was also shown a relationship of CAVI with the presence of obstructive coronary artery disease during CT angiography [12]. In this study revealed that, CAC grade and severe coronary stenosis showed a significant correlation with CAVI. In our study, we failed to identify associations of CAVI with the number of affected coronary arteries, apparently due to more pronounced lesion to the coronary and peripheral arteries.

On the other hand, an increase in the arterial wall stiffness is associated with an increase in afterload on the left heart, which can lead to corresponding morphological changes [3]: an increase in myocardial mass [13,14], the left ventricle diastolic dysfunction [15], the development of heart failure with preserved ejection fraction [16]. When examining individuals without overt cardiovascular disease, high CAVI values were associated with abnormal values of total left ventricular global longitudinal strain in speckle tracking analysis [17]. In the same group of participants, it was noted that the CAVI index was independently associated with the left atrium phase function [18]. The data of the present study on the independent association of CAVI with the left atrium dimention are quite consistent with the above results.

In addition to prognostic value, the assessment of CAVI in CAD patients has another important aspect. The dynamic assessment of this index provides additional opportunities. A recent study suggested that the rapid rise in CAVI in people with high baseline CAVI might be a prodrome of serious cardiovascular events. That is, this factor can mediate the influence of stressful situations on the risk of developing cardiovascular complications [19]. On the other hand, in the absence of a decrease after six months of the initially elevated CAVI in CAD patients, the incidence of cardiovascular events after 2.9 years was significantly higher than in the group with its decrease [7]. Thus, the possibility of improving CAVI in CAD patients has been shown, as well as the possibility of monitoring CAVI in secondary prevention programs in this patients.

Also, subgroup analysis of the TOXO-LIP study showed that a decrease in CAVI during the first year was positively correlated with the development of MACE during five years of follow-up and tended to be an independent predictor of MACE (P = 0.079), this effect was independent of the effect of lowering LDL cholesterol [21]. It remains unclear to what extent changes in CAVI over time may affect the prognosis. A Cardiovascular Prognostic Coupling study is currently underway in Japan to determine the effect of baseline CAVI and changes in CAVI on cardiovascular events [20]. Follow-up is planned for seven years, with an estimate of the time before the onset of a serious cardiovascular event. Data from this registry should provide information on the significance of baseline CAVI and changes in CAVI as indicators of cardiovascular prognosis in patients with at least one risk factor [20]. The significance of such changes in CAVI in patients after CABG remains unexplored, this, apparently, is the task of further research.

### Study limitations

Information about the five-year follow-up was obtained in only 2/3 of patients, which could potentially lead to bias in the study results. However, firstly, we additionally analyzed the patients data who dropped out of the study, among them the percentage of pathological CAVI was comparable to patients with five-year follow-up data, that is, the very fact of the pathological CAVI presence could not affect the outcomes. Secondly, when comparing patients with pathological and normal CAVI among the dropped out patients, it can be noted that for a number of indicators in the pathological CAVI group, patients had more pronounced deviations (bypass duration, heart failure stage, and left ventricular end diastolic dimension) that could adversely affect the prognosis. That is, the inclusion of these patients in the analysis would rather strengthen the association of pathological CAVI with an unfavorable prognosis, than counterbalance it.

In our study, there is no information on the patients adherence to the therapy recommended upon discharge from the hospital after CABG. Nevertheless, all patients were followed up at the place of residence by a cardiologist and, in this respect, were in equal conditions, regardless of preoperative factors and CAVI values.

Also we did not use a number of additional markers of diastolic function, such as tissue Doppler imaging scores, which could strengthen the identified association of CAVI with left ventricular diastolic function based on analysis of LA dimensions. Since the study design did not provide for an extended protocol for assessing left ventricular diastolic function.

We did not evaluate CAVI over time, which does not allow us to understand whether there is the CAVI changes effect on the prognosis in patients after CABG. There is no doubt that subsequent research should be conducted in this direction.

We had to exclude a significant number of patients (with rhythm disturbances, low ejection fraction, low ABI values, valvular pathology) to accurately assess the CAVI relationship with prognosis. Therefore, the CAVI prognostic value is limited only this patients sample and cannot be extended to all CAD patients undergoing coronary artery bypass grafting.

Despite the above limitations, we believe that the fact of identifying the CAVI unfavorable prognostic value in this CAD patient’s cohort after CABG further confirms the clinical usefulness of this index and the prospects for further studies in this direction.

## 5. Conclusion

This study shows that patients with coronary artery disease with the presence of pathological CAVI before CABG surgery within a subsequent five-year follow-up are more likely to develop cardiovascular complications and cardiovascular death than in patients with normal CAVI values. Abnormal CAVI and the number of coronary bypass grafts were independent determinants of the composite endpoint development at follow-up. Age and left atrial dimension were independent factors associated with baseline CAVI. Evaluation of CAVI after CABG in dynamics deserves further study, it is important for monitoring the effects of secondary prevention and the possibility of influencing the prognosis.

## Additional File

The additional file for this article can be found as follows:

10.5334/gh.1053.s1 Supplementary File 1. Tables S1–S4 and Figure S1.
