# Supplementary material for: Assessment of Arterial Stiffness by Cardio-Ankle Vascular Index for Prediction of Five-Year Cardiovascular Events After Coronary Artery Bypass Surgery
Source: Glob Heart. 2021 Dec 27;16(1):90. doi: 10.5334/gh.1053 (PMC8719478; doi:10.5334/gh.1053)
Supplement: Supplementary File 1. — Tables S1–S4 and Figure S1. [file gh-16-1-1053-s1.pdf]

Suppl. Table 1. Baseline patients characteristics not included in the study (n = 118).

Variables	Group 1<br>CAVI <9.0<br>n=79	Group 2<br>CAVI ≥9.0<br>n=39	p
Age, years	55.0(52.0;58.0)	60.0(54.0;70.0)	0.0004
Male sex (n, %)	67(84.81)	28(71.79)	0.065
Height (cm)	172.0 (167.5;177.0)	168.0(162.0;176.0)	0.044
Weight (kg)	80.0(68.0;91.0)	78.5(72.0;90.0)	0.906
BMI (kg/m2)	27.72(24.52;31.1)	28.73(26.23;31.64)	0.154
Smoking (n, %)	39 (49.38)	13(33.33)	0.087
Myocardial infarction history (n, %)	51(64.56)	27(69.23)	0.677
Hypertension (n, %)	66(83.54)	37(94.87)	0.107
Stroke history (n, %)	3(3.79)	4(10.26)	0.168
Transient Ischemic attack (n, %)	1(1.27)	0	0.477
Diabetes mellitus (n, %)	15(18.99)	8(20.51)	0.869
PCI history (n, %)	7(8.86)	3(7.69)	0.815
CABG history (n, %)	0	0	-
Carotid endarterectomy (n, %)	0	1(2.56)	0.155
AP no (n, %)	14 (17.72)	2(5.13)	0.05
AP I functional class (n, %)	3(3.79)	0	0.211
APII functional class (n, %)	24 (30.38)	15(38.46)	0.432
AP III functional class (n, %)	34(43.01)	21(53.85)	0.323
AP IV functional class (n, %)	2(2.53)	1(2.56)	0.991
HF NYHA I (n, %)	56(71.79)	18(46.15)	0.006
HF NYHA II (n, %)	18(23.08)	17(43.59)	0.022
HF NYHA III (n, %)	2(2.53)	0	0.313
Surgical procedure			
Cardiopulmonary bypass (n, %)	67(84.81)	35 (89.74)	0.557
Bypass graft number	2.0(2.0;3.0)	3.0(2.0;3.0)	0.014
Cardiopulmonary bypass duration (min)	89.5(70.0;108.0)	100.0(90.0;118.0)	0.048
Operation duration (min)	240.0(198.0;267.0)	249.0(240.0;300.0)	0.072
Ventriculoplasty, n (%)	5(6.41)	3 (7.69)	0.795
Thrombectomy, n (%)	3(3.85)	2(5.13)	0.746
Radiofrequency ablation, n (%)	3 (3.85)	0	0.214
Carotid endarterectomy, n (%)	2(2.56)	0	0.313
Preoperative echocardiogram			

EDD (cm)	5.5 (5.1;6.0)	5.7(5.4;6.4)	0.042
ESD (cm)	3.8(3.4;4.4)	3.9(3.5;5.1)	0.116
EDVI (ml)	150.0(130.0;180.0)	167.0(150.0;208.0)	0.012
LVESV (ml)	60.0 (46.5;83.0)	66.0(51.0;112.0)	0.153
LA (mm)	4.2(3.8;4.4)	4.3(4.0;4.5)	0.114
LVEF (%)	60.5(51.0;65.0)	60.0(50.0;64.0)	0.713
LVMI	154.1(126.8;182.9)	165.3(130.6;209.6)	0.243
E cm/s	55.5(0.8;68.0)	47.0(0.79;61.0)	0.311
A cm/s	62.0(1.0;75.0)	60.0(0.69;78.0)	0.936
E/A ratio	0.8(0.7;1.2)	0.78(0.69;1.05)	0.289
DT m/s	200.0(169.5;221.0)	200.0(198.0;260.0)	0.306
IVRT m/s	100.0(90.0;109.0)	99.5(88.0;114.5)	0.765
Laboratory data			
Total cholesterol (mmol/L)	5.15(3.65;6.15)	5.4(4.8;6.9)	0.209
HDL cholesterol (mmol/L)	1.22(1.0;1.47)	1.31(0.86;1.7)	0.861
LDL cholesterol (mmol/L)	2.99(2.16;4.1)	3.47(2.36;3.98)	0.516
Triglycerides (mmol/L)	1.49 (1.0;2.13)	1.7(1.34;2.65)	0.143
atherogenic index	3.2(2.1;4.2)	3.3(2.8;4.6)	0.491
Creatinine (μmol/L)	74.0(64.0;91.0)	73.0(62.0; 86.0)	0.778
Glucose (mmol/L)	5.55(5.1;6.2)	5.9 (5.1; 7.2)	0.479
Coronary angiography			
1-coronary artery disease (n, %)	13(16.46)	8(20.51)	0.609
2-coronary artery disease (n, %)	22(27.85)	11(28.21)	0.996
3-coronary artery disease (n, %)	33(41.77)	18(46.15)	0.692
LMCA≥50% (n, %)	12(15.19)	4 (10.26)	0.446
Lesion of non-coronary arteries			
Carotid artery stenosis ≥30% (n, %)	9(11.39)	8(20.51)	0.194
Carotid artery stenosis ≥50% (n, %)	10(12.66)	8(20.51)	0.276
Carotid artery stenosis on both sides ≥30% (n, %)	11 (13.92)	5 (12.82)	0.849
Stenosis of the lower extremities arteries ≥30% (n, %)	9(11.39)	3(7.69)	0.518
Stenosis of the lower extremities arteries ≥50% (n, %)	5(6.33)	3(7.69)	0.795
Stenosis of the lower extremities arteries on both sides ≥30% (n, %)	7(8.86)	3(7.69)	0.815
CIMT (mm)	1.1(1.0;1.2)	1.1(1.0;1.2)	0.135

NOTE. Continuous data are presented as median (lower quartile, upper quartile).

Abbreviations: EDD - end-diastolic dimension; ESD end-systolic dimension; DT - deceleration time; IVRT - isovolumic relaxation time; LVEDV left ventricular end-diastolic volume; LV ESV - left ventricular end-systolic volume; LA - left atrium; CABG - coronary artery bypass graft; LV EF-left ventricular ejection fraction; NYHA- New York Heart Association; PCI - percutaneous coronary intervention; BMI - body mass index; LDL-low-density lipoproteins; CIMT – carotid intima-media thickness; HDL – high-density lipoproteins; LVMI - left ventricular mass index; LMCA - left main coronary artery.

Suppl. Table 2. Linear regression analysis (stepwise method) for the relationship of CAVI with other variables (Coefficients)[a]

Model	Unstandardized Coefficients		Standardized Coefficients	t	Sig.
	B	Std. Error	Beta		
(Constant)	2.835	3.119		0.909	0.364
Male sex	0.124	0.270	0.034	0.458	0.647
Age	0.070	0.013	0.364	5.310	0.000
BMI	-0.032	0.027	-0.083	-1.179	0.240
Myocardial infarction history	-0.126	0.204	-0.039	-0.615	0.539
AP no	-0.209	0.271	-0.052	-0.773	0.440
AP I functional class	0.355	0.627	0.034	0.567	0.571
AP II functional class	0.209	0.213	0.064	0.984	0.326
AP IV functional class	0.118	0.565	0.013	0.208	0.835
HF NYHA I	-0.445	0.407	-0.143	-10.094	0.275
HF NYHA II A	-0.091	0.209	-0.056	-0.436	0.663
HF NYHA II A	-0.165	0.209	-0.058	-0.793	0.429
CABG history	0.260	1.029	0.014	0.252	0.801
PCI history	-0.254	0.347	-0.043	-0.730	0.466
Stroke history	-0.544	0.374	-0.085	-10.452	0.148
Transischemic attack	-0.534	0.863	-0.036	-0.618	0.537
Carotid endarterectomy	-0.233	0.740	-0.018	-0.315	0.753
Hypertension	0.306	0.275	0.066	10.112	0.267
Smoking	0.084	0.219	0.025	0.382	0.703
Diabetes mellitus	0.440	0.283	0.107	10.552	0.122
LA	0.647	0.171	0.247	3.777	0.000

ESD	0.058	0.313	0.033	0.185	0.854
EDD	0.222	0.266	0.116	0.836	0.404
ESV	-0.003	0.009	-0.095	-0.403	0.687
EDV	-0.005	0.006	-0.162	-0.833	0.406
LVEF	-0.019	0.020	-0.133	-0.933	0.352
Carotid artery stenosis ≥30%	0.487	0.282	0.113	1.730	0.085
Carotid artery stenosis ≥50%	0.337	0.338	0.075	0.996	0.320
Carotid artery stenosis on both sides ≥30%	-0.400	0.359	-0.085	-1.113	0.267
CIMT	0.719	0.577	0.078	10.247	0.214
LVMI	-8.521E-6	0.000	-0.043	-0.744	0.457
Glucose	0.008	0.066	0.008	0.116	0.907
Creatinine	-0.002	0.005	-0.022	-0.356	0.722
Total cholesterol	0.044	0.125	0.041	0.347	0.729
LDL cholesterol	-0.011	0.125	-0.009	-0.087	0.931
HDL cholesterol	0.094	0.354	0.020	0.264	0.792
Triglycerides	-0.075	0.108	-0.052	-0.695	0.488
Atherogenicity index	-0.058	0.072	-0.077	-0.815	0.416
Stenosis of the arteries of the lower extremities ≥30%	0.158	0.488	0.030	0.323	0.747
Stenosis of the arteries of the lower extremities ≥50%	-0.697	0.630	-0.100	-1.107	0.269
Stenosis of the arteries lower extremities on both sides ≥30%	-0.058	0.540	-0.012	-0.108	0.914
LMCA≥50%	0.258	0.238	0.062	1.085	0.279
1-coronary artery disease	0.571	0.417	0.148	1.367	0.173
2-coronary artery disease	0.442	0.199	0.262	2.220	0.027
3-coronary artery disease	0.276	0.128	0.267	2.153	0.032
a. Dependent Variable: CAVI					

Abbreviations: AP - angina pectoris; EDD end-diastolic dimension; ESD end-systolic dimension; LVEDV - left ventricular end-diastolic volume; LVESV - left ventricular end-systolic volume; LA - left atrium; CABG - coronary artery bypass graft; LVEF - left ventricular ejection fraction; NYHA - New York Heart Association; PCI - percutaneous coronary intervention; BMI - body mass index; LDL - low-density lipoproteins; CIMT – carotid intima-media thickness; HDL – high-density lipoproteins; LVMI - left ventricular mass index; LMCA - left main coronary artery.

Suppl. Table 3. Linear regression analysis (stepwise method) for the relationship of CAVI with other variables (Model Summary)

Model	R	R Square	Adjusted R Square	Std. Error of the Estimate
1	0.421[a]	0.177	0.174	1.40235
2	0.478[b]	0.228	0.223	1.36080
a. Predictors: (Constant), Age				
b. Predictors: (Constant), Age, left atrium				

Suppl. Table 4. Linear regression analysis (stepwise method) for the relationship of CAVI with other variables (ANOVA)[c]

Model		Sum of Squares	df	Mean Square	F	Sig.
1	Regression	114.580	1	114.580	58.263	0.000[a]
	Residual	530.979	270	1.967		
	Total	645.560	271			
2	Regression	147.430	2	73.715	39.808	0.000[b]
	Residual	498.130	269	1.852		
	Total	645.560	271			

a. Predictors: (Constant), Age

b. Predictors: (Constant), Age, left atrium

c. Dependent Variable: CAVI

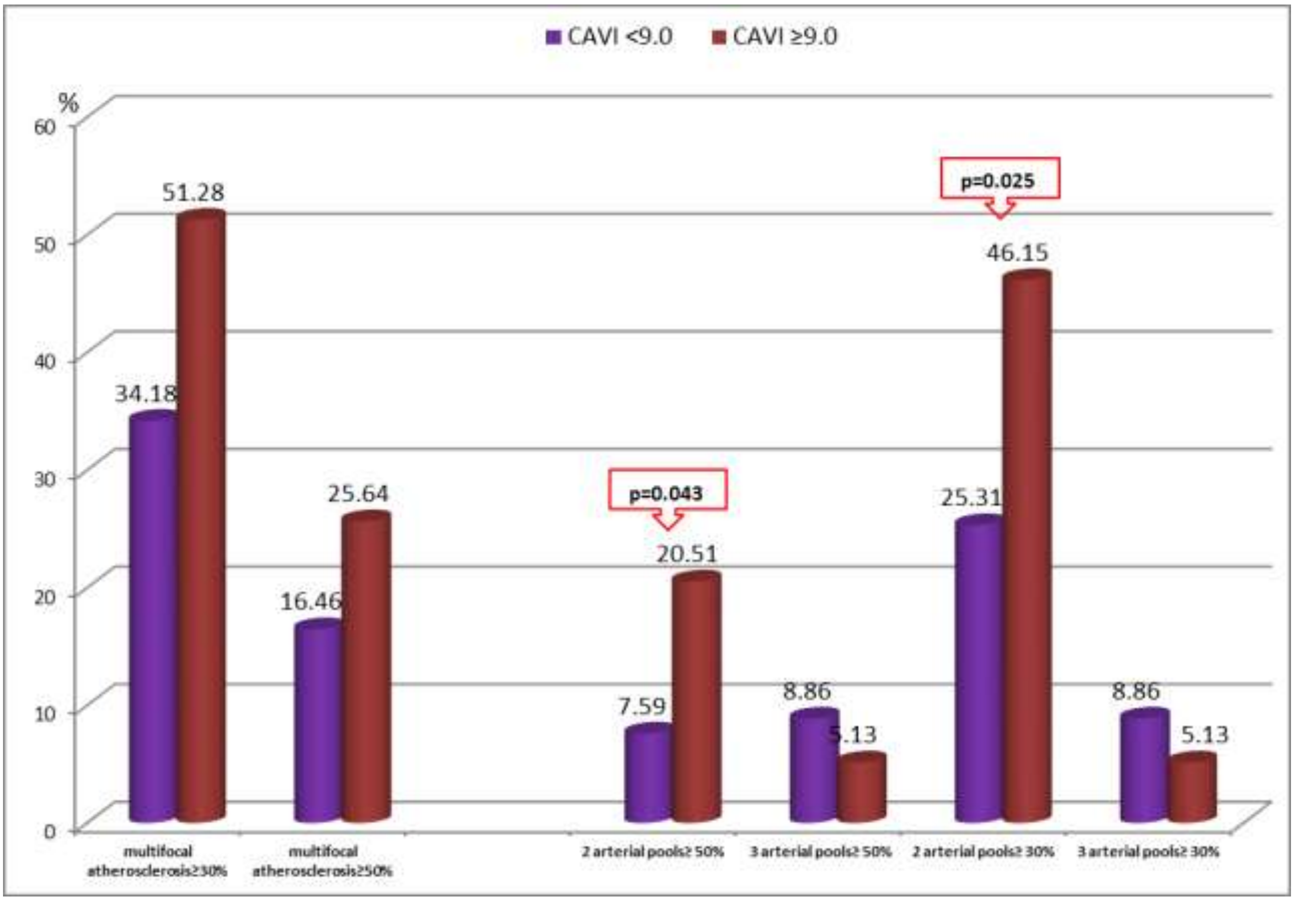

Suppl. Figure 1. Prevalence of multifocal atherosclerosis in groups with abnormal and normal CAVI
